# On estimating ICD-11 and DSM-5-TR prolonged grief disorder prevalence

**DOI:** 10.1017/S0033291726105649

**Published:** 2026-09-03

**Authors:** Maarten C. Eisma

**Affiliations:** Department of Clinical Psychology and Experimental Psychopathology, Faculty of Behavioural and Social Sciences, University of Groningen, Groningen, the Netherlands

Dear Editor-in-Chief,

With interest, I have read the systematic review and meta-analysis by Li et al. (2026). The unique aim of their review was to estimate the prevalence of prolonged grief disorder (PGD) in people bereaved during the COVID-19 pandemic by ‘exclusively including research utilizing ICD-11 and DSM-5-TR standardized assessment tools to enhance diagnostic accuracy’ (p. 2). I commend the authors for their work, because accurate prevalence rates can inform health care provision and policy in future pandemics. Nevertheless, I have a substantive concern about a methodological choice made by Li et al. (2026) that undermines the central aim of their review. Additionally, I will argue that, in light of prior prevalence research, their results should be interpreted much more cautiously.

My main concern is that Li et al. (2026) did not exclusively select studies that used standardized assessment instruments for ICD-11 and DSM-5-TR PGD, thereby diverging from their primary aim. ICD-11 and DSM-5-TR PGD were established only recently (in 2019 and 2022, respectively) and show limited semblance to several previously proposed grief disorders, including the 2009 proposal for PGD, a 2016 proposal for PGD for ICD-11, and Persistent Complex Bereavement Disorder (PCBD), included for further study in the DSM-5 (for an overview, see Eisma, 2023). Current diagnoses differ from past proposed disorders and each other in symptom count and content (overlap is moderate), (the existence of) diagnostic algorithms, and time since loss criteria, that is, either 6 or 12 months post-loss (Eisma, 2023). Therefore, phenomenological characteristics of disordered grief, such as prevalence, may vary depending on which criteria sets are applied (e.g. Boelen & Lenferink, 2020). Similarly, measurement instruments to assess symptoms of grief disorders developed before the establishment of ICD-11 and DSM-5-TR PGD (for a review, see Treml, Kaiser, Plexnies, & Kersting, 2020) do not correspond to current criteria sets and therefore likely yield different findings than instruments specifically developed to assess the most recent PGDs.

To illustrate, the Prolonged Grief 13 (PG-13) does not cover one core criterion (i.e. cognitive preoccupation) and two accessory criteria from DSM-5-TR PGD (i.e. disbelief and loneliness). Similarly, the Traumatic Grief Inventory Self-Report (TGI-SR) does not cover two accessory criteria of PGD DSM-5-TR (i.e. disbelief and identity disruption) and four accessory criteria of PGD ICD-11 (i.e. denial, blame, feeling a part of self has died, and inability to experience positive mood). Moreover, many older instruments, particularly the Inventory of Complicated Grief (ICG) and the related Inventory of Complicated Grief Revised (ICG-R), measure many symptoms that are not included in DSM-5-TR and ICD-11 PGD. For example, the ICG-R has 30 items, while there are only 10 core and accessory DSM-5-TR PGD symptoms and 12 core and accessory ICD-11 PGD symptoms. Therefore, cutoffs derived from outdated instruments, such as the PG-13, TGI-SR, ICG, and ICG-R, should, in principle, not be used to estimate the prevalence of ICD-11 and DSM-5-TR PGD. This implies that estimates from 6 of 13 studies (46%) included in the meta-analysis by Li et al. (2026) that applied such measures are irrelevant to the central aim of the study. I recommend that the authors provide prevalence estimates using only results from studies that have applied instruments developed to assess ICD-11 and DSM-5-TR PGD symptomatology, such as the International Prolonged Grief Disorder Scale (IPGDS) (Killikelly et al., 2020) or the Traumatic Grief Inventory Self-Report Plus (TGI-SR+) (Lenferink et al., 2022).

Additionally, more guidance should be provided by Li et al. (2026) to help interpret their results, which suggest that about a quarter of people bereaved during the pandemic developed PGD. Notably, their estimated prevalence rates are six- to eightfold higher than those observed in representative bereaved population samples, which show that probable ICD-11 and DSM-5-TR PGD is experienced by 3–4 percent of bereaved adults (e.g. Rosner, Comtesse, Vogel, & Doering, 2021). Li et al.’s (2026) results appear alarming, given that globally over 120 million people died during the pandemic due to any cause, corresponding to a much larger number of bereaved persons. Yet, when interpreting findings, Li et al. (2026) simply state that there is ‘a high pooled prevalence of PGD observed during the COVID-19 pandemic’ (p. 11) … ‘exceeding most prevalence ranges reported in pre-pandemic works’ (p. 8), leading them to conclude that ‘structural traumas, e.g., restricted access at the deathbed and disruptions to traditional mourning practices, … may prolong grief pathology beyond typical acute stress responses’ (p. 9). Apart from the fact that this statement inflates the concept of trauma and incorrectly suggests that acute grief following bereavement is pathological, it does not offer detailed considerations on how their dataset may explain these exceptionally high pooled prevalence estimates of PGD.

I believe that the estimated prevalence of PGD derived by Li et al. (2026) is likely an overestimation, because at least 10 of the 13 studies included in this review used convenience sampling. A comprehensive review of 24 studies on PGD prevalence has shown that such nonprobability sampling gives three times higher observed prevalence rates than probability sampling (16% vs. 5%) (Comtesse et al., 2024). It seems that people who are more severely affected by bereavement are more likely to voluntarily participate in grief research, potentially because it is more personally relevant to them, which biases estimated prevalence (Eisma, 2025). Additionally, the Chinese sample that gives the highest PGD prevalence estimates, possibly because it consists exclusively of adults bereaved due to COVID-19, appears to be included in the meta-analysis twice, further inflating pooled prevalence estimates.

In conclusion, the meta-analysis by Li et al. (2026) failed to deliver on its primary aim, because close to half of the included studies did not use validated instruments of ICD-11 and DSM-5-TR PGD. Moreover, the predominant use of voluntary response sampling and double counting of a sample reporting high PGD symptom levels implies that the pooled prevalence rates of PGD derived from this meta-analysis are overestimations. To prevent false alarms about PGD among the general bereaved population during the COVID-19 pandemic, I advise to be very cautious when interpreting the results from this meta-analysis.
